# MRI characteristics of cervical radioiodine-avid lymph nodes detected on post-therapeutic ¹³¹I whole-body scintigraphy in differentiated thyroid carcinoma

**DOI:** 10.3389/fendo.2026.1804633

**Published:** 2026-06-23

**Authors:** Liang-Qian Tong, Zhuo-Wen Li, Wei Liu, Yan-Fang Sui, Yue Chen

**Affiliations:** 1 Department of Nuclear Medicine, The Affiliated Hospital of Southwest Medical University, Luzhou, Sichuan, China; 2 Nuclear Medicine and Theranostics Key Laboratory of Sichuan Province, Luzhou, Sichuan, China; 3 Institute of Nuclear Medicine, Southwest Medical University, Luzhou, Sichuan, China; 4 Department of Nuclear Medicine, Haikou Research Institute of Xiangya School of Medicine, Central South University, Haikou, Hainan, China; 5 Department of Rehabilitation Medicine, Haikou Research Institute of Xiangya School of Medicine, Central South University, Haikou, Hainan, China

**Keywords:** 131I therapy, differentiated thyroid carcinoma (DTC), imaging characteristics, lymph node metastasis, MRI

## Abstract

**Objective:**

This study aimed to characterize the magnetic resonance imaging (MRI) features of cervical metastatic lymph nodes in differentiated thyroid carcinoma (DTC) and provide imaging-based evidence for identifying metastatic cervical lymph nodes prior to administration of radioactive iodine (¹³¹I) therapy.

**Methods:**

Clinical and imaging data from 32 patients with histologically confirmed DTC who underwent ¹³¹I therapy at Haikou People’s Hospital between June 2019 and May 2024 were retrospectively analyzed. All patients demonstrated cervical radioiodine-avid lymph nodes on post-therapeutic ¹³¹I whole-body scintigraphy (¹³¹I-Rx-WBS). Cervical ultrasonography (US) and MRI examinations were conducted prior to ^131^I therapy. The MRI characteristics of the lymph nodes identified as radioiodine-avid on ¹³¹I-Rx-WBS were assessed, including anatomical location, number, shape, margin definition, presence of hilum, proximity to vascular structures, and relative size within the nodal population.

**Results:**

All participants were diagnosed with papillary thyroid carcinoma. The median age was 45.5 years (range, 28–66 years). Cervical US detected lymph node metastasis in 15.6% (5/32) of cases. A total of 58 radioiodine-avid lymph nodes were identified across cervical levels II–VI and the supraclavicular region. MRI analysis demonstrated that most radioiodine-avid lymph nodes exhibited hyperintensity on T_1_- and T_2_-weighted sequences. Morphologically 65.5% were round to ovoid and 32.8% were irregular in shape. Ill-defined margins were observed in 69.0% of nodes, absence of the lymphatic hilum in 91.4%, and vascular adjacency in 77.6%. In 96.9% (31/32) of patients, at least 4 lymph nodes were visualized on MRI, however, imaging features of radioiodine-negative lymph nodes often overlapped with those of radioiodine-avid nodes.

**Conclusion:**

Cervical lymph nodes demonstrating radioiodine uptake on ¹³¹I-Rx-WBS frequently present with round to ovoid or irregular morphology and indistinct margins on MRI. However, given the high density of cervical lymph nodes and overlapping imaging characteristics between radioiodine-avid and radioiodine-negative nodes, a comprehensive assessment integrating multimodal imaging and clinical correlation is essential for accurate assessment before ¹³¹I therapy.

## 1 Introduction

Differentiated thyroid carcinoma (DTC) represents the most prevalent form of thyroid malignancy, accounting for over 90% of all thyroid cancer cases, with papillary thyroid carcinoma constituting the predominant histopathological subtype (1, 2). Although DTC is generally associated with a favorable prognosis, the presence of cervical lymph node metastasis increases the risk of locoregional recurrence and distant metastasis, and is significantly associated with reduced survival outcomes (3, 4).

Postoperative administration of radioactive iodine (¹³¹I) remains a cornerstone in the management of DTC, facilitating the elimination of residual thyroid tissue and metastatic lesions. Accurate identification of cervical lymph node metastases is fundamental to optimizing ¹³¹I treatment planning (5). However, reliable pre-therapeutic evaluation of metastatic lymph node remains a significant clinical challenge.

In current clinical practice, cervical ultrasonography (US) is the preferred initial imaging modality for evaluating lymph node involvement in DTC. Its diagnostic performance is constrained by operator experience and limited visualization of deep or anatomically inaccessible lymph nodes, with false-negative rates exceeding 30% (3, 6). Post-therapeutic ¹³¹I whole-body scintigraphy (¹³¹I-Rx-WBS) enables functional assessment through detection of iodine-avid lesions; however, its spatial resolution is suboptimal (typically > 1 cm), thereby hindering the accurate localization of small or non–iodine-avid lymph nodes (7).

Magnetic resonance imaging (MRI), owing to its superior soft-tissue resolution and multiparametric capabilities, has gained increasing attention as a complementary imaging modality for assessing cervical lymph node metastases. Nevertheless, MRI-based characterization of metastatic lymph nodes remains inconsistent. Certain studies have reported that round morphology, absence of the lymphatic hilum, and vascular adjacency are characteristic of metastatic involvement (8); however, similar features may also be observed in benign lymph nodes, resulting in diagnostic ambiguity and false positive findings (9).

The correlation between iodine-avid lymph nodes identified on ¹³¹I-Rx-WBS and their corresponding MRI characteristics has not been systematically investigated, limiting the pre-therapeutic diagnostic utility of MRI. Therefore, the present study retrospectively analyzed MRI characteristics of cervical lymph nodes exhibiting radioiodine avidity on ¹³¹I-Rx-WBS in patients with DTC. The aim is to clarify the diagnostic value of these imaging features and provide evidence to support clinical decision-making in preparation for ¹³¹I therapy. Specifically, accurate pre-therapeutic identification of cervical lymph node metastases can guide risk-adapted ¹³¹I therapy. According to the American Thyroid Association (ATA) guidelines (2), the presence of nodal metastases may warrant a higher administered activity (e.g., 150 mCi vs. 30–100 mCi) or modified treatment strategy to improve disease-free survival. Thus, MRI detection of suspicious nodes before ¹³¹I administration could facilitate individualized treatment planning.

## 2 Materials and methods

### 2.1 Study population

This retrospective study initially screened 187 consecutive patients with histologically confirmed DTC who underwent total thyroidectomy followed by ¹³¹I therapy at the study institution between June 2019 and May 2024.

Inclusion criteria were: (1) presence of cervical radioiodine-avid lymph nodes on post-therapeutic ¹³¹I whole-body scintigraphy (¹³¹I-Rx-WBS); (2) complete cervical MRI and US examinations performed before ¹³¹I administration; (3) available clinical and pathological data.

Exclusion criteria were: (1) absence of radioiodine-avid cervical lymph nodes on ¹³¹I-Rx-WBS; (2) incomplete pre-therapeutic MRI or US data; (3) poor image quality precluding accurate node evaluation; (4) previous external beam radiotherapy to the neck.

**Figure 1:**
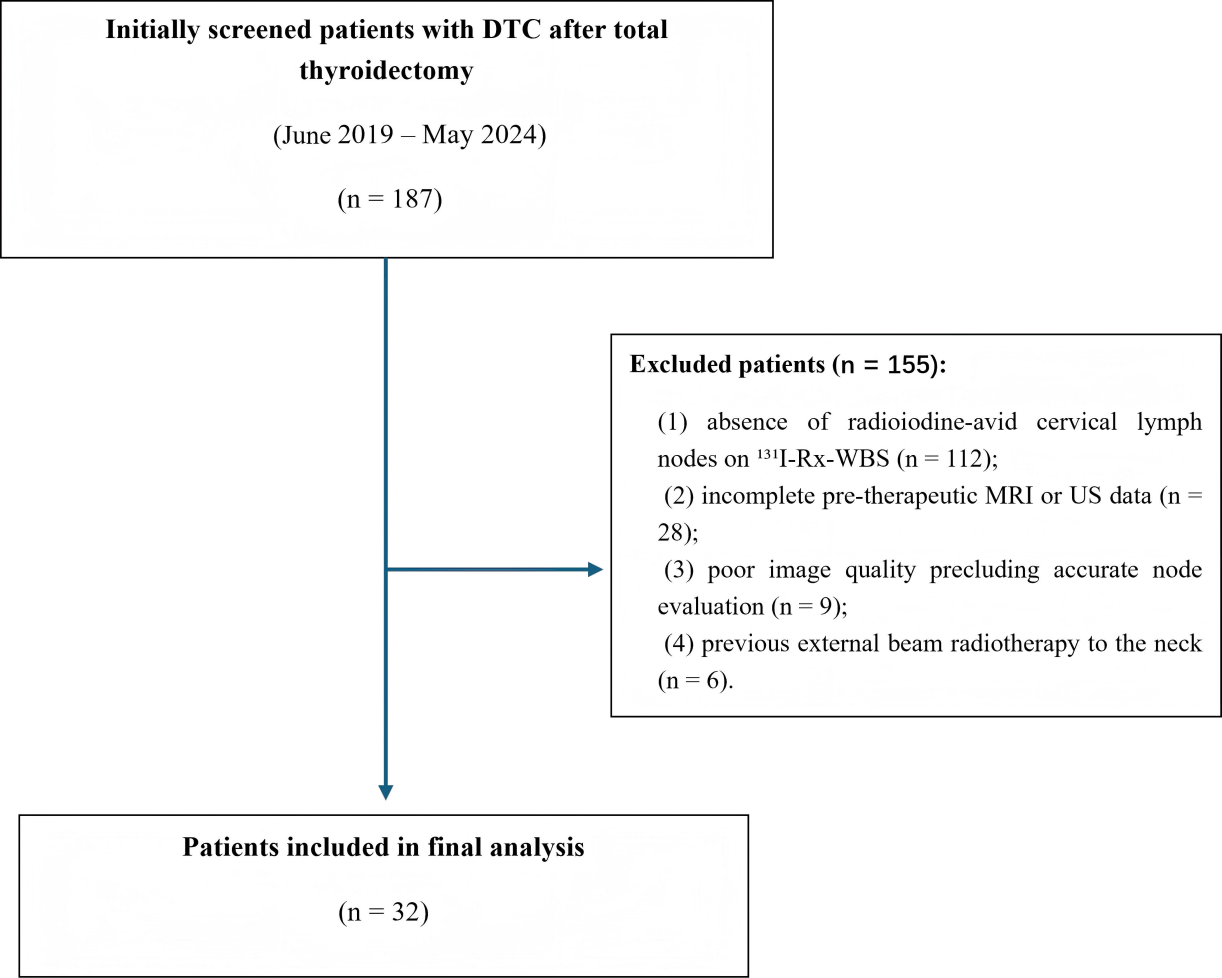
Flowchart of patient selection. From 187 initially screened patients with DTC after total thyroidectomy, 155 were excluded for the following reasons: no radioiodine-avid cervical lymph nodes on ¹³¹I-Rx-WBS (n=112), incomplete pre-therapeutic MRI/US data (n=28), poor image quality (n=9), or prior neck radiotherapy (n=6). The remaining 32 patients met all inclusion criteria and were analyzed.

After applying these criteria, 32 patients were included in the final analysis. All cases were histopathologically confirmed as DTC following total thyroidectomy, which had been performed at least 1 month before study enrollment. After enrollment, patients underwent pre-therapeutic evaluation (cervical US and MRI) prior to ¹³¹I administration, followed by post-therapeutic whole-body scintigraphy on day 3 after ¹³¹I therapy. The patient selection process is summarized in **Figure 1**.

### 2.2 Imaging protocols

#### 2.2.1 Post-therapeutic ^131^I-Rx-WBS

Post-therapeutic ¹³¹I-Rx-WBS was performed on day 3 following ¹³¹I administration using a GE SPECT/CT system (Infinia Hawkeye 4). Acquisition parameters included a high-energy general-purpose collimator, with a photopeak centered at 364 keV and a 20% energy window. Whole-body scans were acquired at a speed of 10 cm/min, and a matrix size of 256 × 256. Images were processed on a Xeleris 3 workstation and independently reviewed by two nuclear medicine physicians. Lymph nodes were classified as radioiodine-avid if the tumor-to-normal (T/N) uptake ratio exceeded 2.0 or if focal radiotracer accumulation was visually identified (1). This functional criterion was used solely to select nodes for MRI characterization; it does not serve as an independent gold standard for metastasis. Histopathological confirmation was not available for these nodes because invasive biopsy in the post-therapeutic setting was neither clinically indicated nor ethically justified. To enable correlation with pre-therapeutic MRI, the anatomical location of each radioiodine-avid lymph node was recorded based on cervical level classification (levels II–VI and supraclavicular region) using SPECT/CT fusion images when available, or by topographic landmarks on planar images. Two nuclear medicine physicians and two radiologists jointly reviewed the images to ensure consistent anatomical matching.

#### 2.2.2 Cervical MRI

MRI of the cervical region was performed 1–3 days before ¹³¹I therapy using a 3.0 Tesla system (GE Healthcare). Imaging sequences included T_1_-weighted imaging (T_1_WI; repetition time [TR]/echo time [TE] = 500/10 ms), T_2_-weighted imaging (T_2_WI; TR/TE = 4000/90 ms), and T_2_-weighted short-tau inversion recovery (T_2_W STIR; TR/TE/inversion time [TI] = 5000/70/150 ms). Images were independently reviewed by two radiologists who were aware of the ¹³¹I-Rx-WBS findings, as the study aimed to characterize the MRI features of known radioiodine-avid lymph nodes. The radiologists specifically evaluated the lymph nodes at anatomical sites corresponding to focal uptake on the post-therapeutic scan. Parameters analyzed included lymph node location, number, shape, margin definition, presence or absence of a lymphatic of hilum, proximity to vascular structures, and identification of the largest node. In addition, for each radioiodine-avid lymph node, the maximal long-axis diameter (L) and the perpendicular short-axis diameter (S) were measured on axial T_2_-weighted images; the long-to-short axis ratio (L/S ratio) was then calculated. All measurements were performed independently by two radiologists, and the average value was used for analysis.

In contrast, cervical US was performed and interpreted prior to ¹³¹I therapy, and the sonographers were blinded to the subsequent scintigraphy results.

### 2.3 Statistical analysis

All statistical analyses were performed using SPSS software, version 22.0 (IBM Corp., Armonk, NY, USA). Continuous variables were expressed as mean ± standard deviation or were summarized using median and range due to the small sample size and the lack of a normal distribution, while categorical variables were expressed as frequencies and percentages. No inferential statistical tests (e.g., t-test, chi-square test) were performed, as this study was purely descriptive in nature, aiming to characterize MRI features without group comparisons.

## 3 Results

### 3.1 Patient characteristics

Of the 32 patients included in the study, 14 were male and 18 were female, with a median age of 45.5 years (range, 28–66 years). All patients were diagnosed with papillary thyroid carcinoma. Tumor focality was unifocal in 50.0% (16/32), bifocal in 25.0% (8/32), and multifocal in 25.0% (8/32). According to the AJCC 8th edition TNM staging system, the distribution of pathological tumor (pT) stages was as follows: pT1 in 8 patients (25.0%), pT2 in 4 patients (12.5%), pT3a in 12 patients (37.5%), pT3b in 6 patients (18.8%), and pT4a in 2 patients (6.2%). Regarding pathological nodal status, all 32 patients had confirmed pN1a or pN1b at the time of thyroidectomy. The median number of surgically resected lymph nodes was 8 (range, 3-18), among which the median number of pathologically confirmed metastatic lymph nodes was 3 (range, 1-7). All patients underwent total thyroidectomy. Lymphadenectomy was performed in all cases (central neck dissection in 100%; additional lateral neck dissection in 14/32 patients, 43.8%). The administered activity of ¹³¹I ranged from 3.7 to 5.55 GBq (100–150 mCi), with a median activity of 4.44 GBq (120 mCi).

### 3.2 Comparison of cervical US and ^131^I-Rx-WBS

Cervical US identified metastatic lymph nodes in 5 patients (15.6%), whereas ¹³¹I-Rx-WBS identified radioiodine-avid cervical lymph nodes in all 32 cases. A total of 58 radioiodine-avid lymph nodes were localized across the following cervical regions: 18 in level II, 8 in level III, 18 in level IV, 1 in level V, 10 in level VI, and 3 in the supraclavicular region.

### 3.3 MRI imaging features

**Figure 2:**
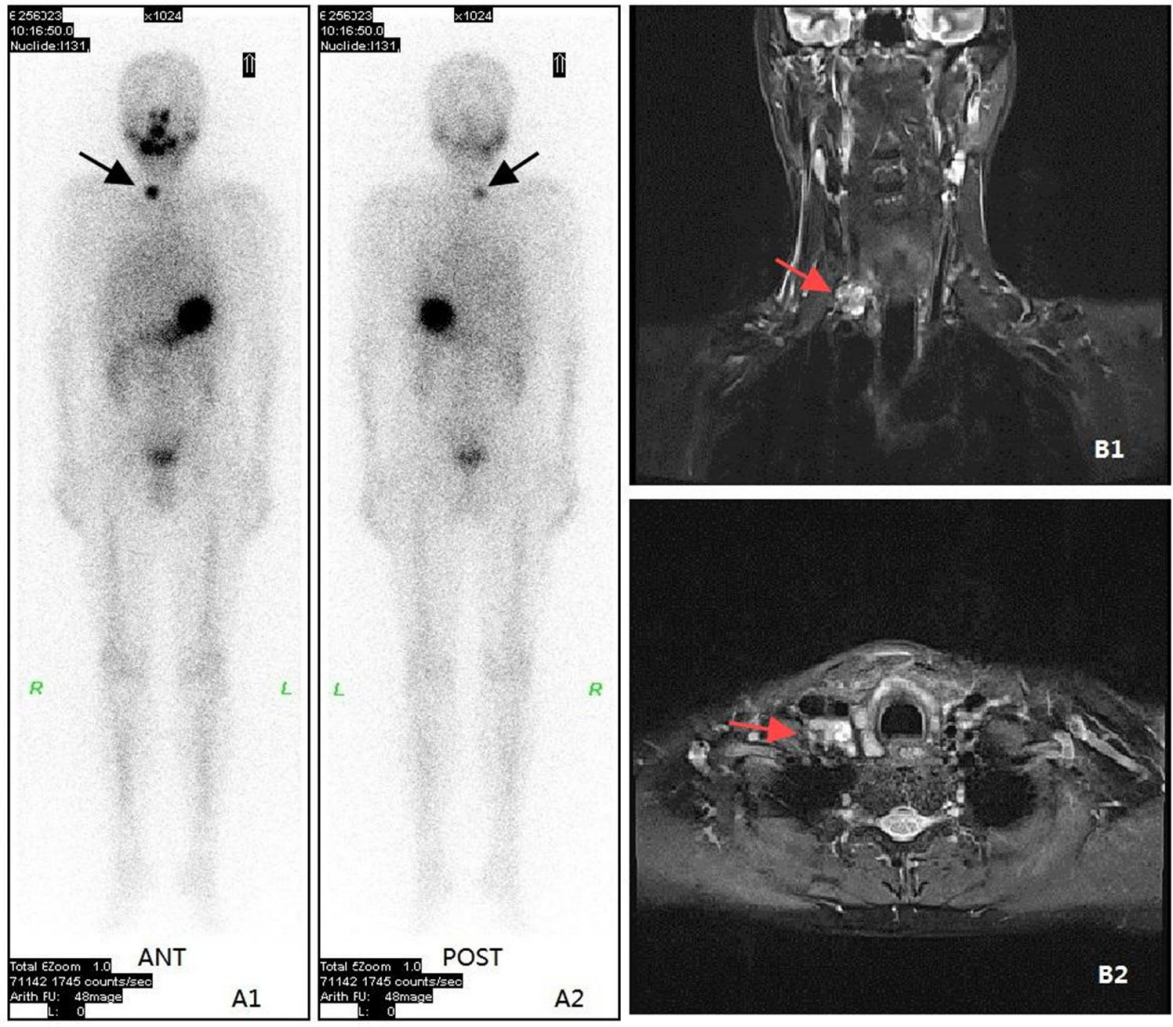
MRI features of lymph nodes positive on ^131^I-Rx-WBS. **(A1, A2)** anterior and posterior ^131^I-Rx-WBS images showing a positive lymph node in the right cervical level IV (black arrow), suggestive of cervical lymph-node metastasis from thyroid carcinoma. **(B1, B2)** coronal and axial fat-suppressed T2-weighted cervical MRI of the same patient; the corresponding positive node in right level IV is irregular in shape, exhibits heterogeneous hyper-intense T2 signal, lies adjacent to a vessel, and shows an indistinct hilum (red arrow).

On MRI, the majority of radioiodine-avid lymph nodes demonstrated prolonged signal intensities on both T_1_- and T_2_-weighted sequences. Morphologically, 65.5% of lymph nodes were round to ovoid, 32.8% were irregular in shape. Ill-defined margins were observed in 69.0% of cases, with absence of the lymphatic hilum in 91.4%, and vascular adjacency in 77.6% of nodes (**Figure 2**).

Quantitative size analysis of the 58 radioiodine-avid nodes revealed: mean short-axis diameter 9.2 ± 3.4 mm (median 8.5 mm, range 4.5-18.0 mm); mean long-axis diameter 13.8 ± 5.1 mm (median 12.5 mm, range 7.0-26.0 mm); and mean L/S ratio 1.52 ± 0.38 (median 1.45, range 1.05-2.80). A round or ovoid shape (L/S ratio < 2.0) was observed in 65.5% (38/58) of nodes, while an L/S ratio > 2.0 (more elongated) was seen in 34.5% (20/58). Notably, in 96.9% (31/32) of patients, at least 4 cervical lymph nodes were visualized on MRI. Among the numerous lymph nodes visible on MRI without corresponding radioiodine uptake, many exhibited similar imaging characteristics, including round morphology, ill-defined margins, absent or indistinct hilum, and proximity to vascular structures. Importantly, due to the lack of a pathological gold standard in this post-therapeutic setting, it could not be determined whether these radioiodine-negative nodes represented benign reactive nodes or non-avid metastatic nodes.

### 3.4 Patient-level correlation of imaging modalities

Of the 32 patients, cervical US identified suspicious metastatic lymph nodes in 5 patients (15.6%). In these 5 patients, ¹³¹I-Rx-WBS confirmed radioiodine-avid lymph nodes in the same cervical levels in all cases, and MRI also demonstrated corresponding nodes with the characteristic imaging features described above.

Conversely, among the 32 patients, ¹³¹I-Rx-WBS detected at least one radioiodine-avid lymph node in every patient. In all 32 patients, MRI was able to visualize a lymph node at the anatomical site corresponding to each radioiodine-avid focus (total 58 nodes). No radioiodine-avid node was identified on ¹³¹I-Rx-WBS that could not be located on MRI, supporting the high anatomical concordance between the two modalities.

Regarding additional lymph nodes visible on MRI but not on ¹³¹I-Rx-WBS: in all 32 patients, MRI visualized a mean of 4.8 (range, 4-10) cervical lymph nodes per patient that showed no corresponding radioiodine uptake. These radioiodine-negative nodes frequently shared similar morphological features with the avid nodes (as described in 2.3), but whether they represented benign reactive nodes or non−avid metastases could not be determined without pathological confirmation.

## 4 Discussion

DTC accounts for over 90% of all thyroid malignancies (1). Despite its favorable prognosis, cervical lymph node metastasis remains a common metastatic pathway and is significantly associated with locoregional recurrence and patient survival (10). Therefore, accurate postoperative identification of metastatic lymph nodes is crucial for risk stratification and treatment planning. While post-therapeutic ¹³¹I whole-body scintigraphy (¹³¹I-Rx-WBS) can detect iodine-avid lesions, its low spatial resolution limits precise anatomical localization. Additional cervical MRI, performed before ¹³¹I therapy, provides superior soft-tissue contrast and multiplanar capabilities, enabling exact determination of nodal size, shape, margins, hilum status, and vascular adjacency. This anatomical precision helps distinguish metastatic from benign nodes and guides individualized ¹³¹I dosing (e.g., higher activity for involved nodes) and surgical planning if needed. Thus, MRI adds clinically relevant value beyond functional scintigraphy alone.

Cervical US is the first-line modality for detecting lymph node metastases in DTC; however, its diagnostic performance is limited, particularly in preoperative and pre-radioiodine therapeutic settings, due to operator dependency and difficulty in detecting deeply situated lymph nodes. As such, false-negative findings are common (11). Although ¹³¹I-Rx-WBS serves as a key tool for identifying postoperative metastatic foci, its limited spatial resolution restricts precise anatomical localization of metastatic lymph nodes (2). These limitations underscore the need for advanced imaging modalities with improved sensitivity and specificity for detecting cervical lymph node metastasis.

The present study evaluated 32 patients with DTC who had undergone surgery and subsequently received ¹³¹I therapy, with the aim of characterizing the MRI features of cervical lymph nodes demonstrating radioiodine-avidity on ¹³¹I-Rx-WBS. The most frequently observed MRI features included round to ovoid (65.5%) or irregular morphology (32.8%), ill-defined margins (69.0%), absence of the lymphatic hilum (91.4%), and adjacency to vascular structures (77.6%). These findings are consistent with previously reported imaging characteristics of metastatic cervical lymph nodes in DTC (12). However, these features were also commonly observed in radioiodine-negative nodes, indicating substantial overlap. This highlights the need for multimodal imaging and clinical data integration to improve diagnostic accuracy (13).

The findings align with those reported in recent studies. Zhang et al. (14) reported that metastatic cervical lymph nodes in DTC frequently exhibit round to ovoid or irregular morphology, ill-defined margins, and absent hila on MRI. Similarly, Qin et al. (15) confirmed the high sensitivity of cervical MRI in the preoperative assessment of lymph node metastases. Nevertheless, neither of these studies systematically analyzed MRI characteristics in relation to ^131^I-Rx-WBS findings. Lee et al. (16) also reported overlapping imaging features between radioiodine-avid and non-avid nodes, further reinforcing the importance of a comprehensive, multimodal diagnostic approach.

The principal contribution of this study lies in its provision of imaging evidence to support the pre-therapeutic identification of cervical metastatic lymph nodes in patients with DTC. Implementing cervical MRI prior to ¹³¹I therapy may facilitate more accurate assessment of nodal status and optimize treatment planning. Moreover, advancements in functional MRI techniques, particularly diffusion-weighted imaging (DWI) and dynamic contrast-enhanced MRI (DCE-MRI), hold promise for further enhancing diagnostic accuracy (17, 18).

This study contributes to radiologic evidence for pre-¹³¹I therapeutic decision-making in differentiated thyroid carcinoma (DTC) by focusing on a targeted analysis: it systematically correlates the MRI features of cervical lymph nodes with their radioiodine avidity on ¹³¹I-Rx-WBS. This design addresses a specific gap in existing research—namely, the lack of detailed data linking functional iodine-avidity (a key marker for DTC metastasis) to structural MRI characteristics—providing clinically relevant insights for identifying metastatic nodes before therapy.

We acknowledge that ¹³¹I uptake is not absolutely specific for lymph node metastasis. Although uncommon in cervical lymph node regions outside the thyroid bed, false-positive uptake can theoretically arise from inflammatory lymph nodes (due to increased perfusion or sodium-iodide symporter expression in activated lymphocytes), ectopic gastric mucosa, thymic tissue, or salivary gland secretion. In our cohort, all 58 radioiodine-avid nodes were located in levels II–VI or the supraclavicular region, which are typical metastatic drainage sites for DTC; no nodes were identified in regions known for physiological pitfalls (e.g., submandibular glands, thymus). Moreover, none of the patients had clinical signs of active cervical infection. Nevertheless, we cannot completely exclude the possibility that a small fraction of these nodes represented false-positive uptake. If such false-positive nodes were inadvertently included, the reported MRI features (e.g., round/irregular shape, ill-defined margins, absent hilum) might slightly overestimate their specificity for metastasis. However, given the high prevalence of true metastasis in this post-thyroidectomy DTC population with elevated thyroglobulin, the overall descriptive findings remain clinically useful.

Despite this value, several limitations of the study must be acknowledged. First, the retrospective design and potential selection bias may affect the robustness of the findings. Second, the study cohort (n = 32) is relatively small, which restricts the generalizability of the observed MRI features to broader DTC populations. Additionally, functional MRI sequences (e.g., DWI and DCE-MRI), which have been shown to improve the diagnostic accuracy of lymph node metastasis, were not incorporated here; future studies integrating these sequences could further refine the imaging assessment of cervical nodes in DTC.

We acknowledge that the statistical analysis in this study is descriptive only, without inferential comparisons between groups. This is because the radioiodine-negative lymph nodes lacked a pathological reference standard, precluding any reliable classification into benign vs. non-avid metastatic nodes. Under these circumstances, comparative statistics (e.g., sensitivity, specificity, odds ratios) would be inappropriate and potentially misleading. Therefore, the present analysis deliberately limits itself to characterizing the MRI features of radioiodine-avid nodes, which serve as a functional gold standard for metastatic involvement.

We acknowledge the concern regarding the absence of a histopathologically confirmed control group. However, in the specific clinical context of patients who have already undergone total thyroidectomy and comprehensive neck dissection, performing invasive biopsies (fine needle aspiration or excisional biopsy) on cervical lymph nodes solely for research validation is neither clinically indicated nor ethically justified. Such procedures carry risks of bleeding, infection, nerve damage, and potential tumor seeding, without any anticipated therapeutic benefit for the patient. Consequently, the field of post therapeutic DTC lymph node imaging has necessarily relied on functional surrogates — most commonly radioiodine avidity on ¹³¹I Rx WBS — as the best available reference standard. This methodological approach is consistent with previous publications. For example, Lee et al. employed a composite reference of ¹³¹I avidity and serial follow up for CT/US comparison (16); and Zhang et al. validated MRI radiomics against ¹³¹I Rx WBS without pathological confirmation of every node (14). Following these established precedents, the present study does not claim to provide diagnostic accuracy metrics (sensitivity, specificity, AUC). Instead, it offers a descriptive morphological ‘atlas’ of radioiodine avid cervical lymph nodes — a necessary foundation for future prospective studies that may incorporate FNA Tg or long term follow up as validation standards.

Despite this limitation, the clinical relevance of our findings is twofold. First, the observed MRI features (round/irregular shape, ill-defined margins, absent hilum, vascular adjacency) provide a morphological ‘signature’ of radioiodine-avid metastases, which can help radiologists and nuclear medicine physicians recognize suspicious nodes on pre-therapeutic MRI. Second, as noted in the Introduction, the presence of nodal metastases justifies a higher ¹³¹I activity (e.g., 150 mCi instead of 30–100 mCi) according to ATA guidelines (2). By detecting such nodes before ¹³¹I therapy, MRI can directly influence risk-adapted dosing. A practical example would be: a patient with MRI-detected round, ill-defined nodes in level IV would be scheduled for 5.55 GBq (150 mCi) rather than 3.7 GBq (100 mCi). Thus, even without advanced statistics, our findings offer actionable information for individualizing treatment.

Given the overlapping features observed between radioiodine-avid and -negative nodes, future research should explore complementary approaches. Quantitative MRI parameters—such as ADC values, T1/T2 relaxation times, and enhancement ratios—should be incorporated and validated against histopathology. Specifically, molecular imaging techniques such as ¹^8^F-FDG PET/CT could be incorporated to detect iodine-negative metastases in patients with elevated thyroglobulin but negative ¹³¹I scan, thereby enabling accurate localization and targeted therapy (19). Simultaneously, AI-assisted systems could be developed to objectively quantify subtle MRI features and build predictive models, potentially improving diagnostic consistency and reducing observer variability. Continued research into these integrated and intelligent imaging strategies is expected to further enhance diagnostic precision and clinical outcomes.

To overcome the lack of a gold standard in the present study, future prospective investigations should consider: (a) ultrasound-guided fine-needle aspiration cytology with thyroglobulin washout (FNA-Tg) for suspicious radioiodine-avid nodes when clinically indicated; (b) SPECT/CT fusion imaging to more precisely localize focal uptake and exclude physiological interference; or (c) longitudinal follow-up with serial thyroglobulin measurements and imaging to confirm the metastatic nature of nodes that remain radioiodine-avid over time. Such validation would enable formal diagnostic accuracy analyses (sensitivity, specificity, predictive values) for individual MRI features and their combinations.

## 5 Conclusion

Cervical MRI offers diagnostic utility in the detection of lymph node metastasis in DTC, particularly in the context of pre-¹³¹I therapeutic evaluation, providing key radiological evidence for clinical decision-making. However, due to the high density of cervical lymph nodes and the substantial overlap in imaging features between radioiodine-avid and radioiodine-negative nodes, accurate pre-therapeutic diagnosis remains challenging. Consequently, comprehensive assessment integrating multimodal imaging approaches and relevant clinical data is essential. Ongoing advancements in MRI technology, particularly the application of functional imaging sequences such as DWI and DCE-MRI, are expected to further improve the sensitivity and specificity of MRI in detecting metastatic cervical lymph nodes in DTC.

## Data Availability

The original contributions presented in the study are included in the article/supplementary material. Further inquiries can be directed to the corresponding authors.
